# Risk Factors for Recurrence after Surgical Resection of Sinonasal Inverted Papilloma

**DOI:** 10.1055/s-0044-1785206

**Published:** 2024-05-25

**Authors:** Eugénie Delaine, François Gorostidi, Pierre Guilcher, Karma Lambercy, Yann Litzistorf, Luc Bron, Antoine Reinhard

**Affiliations:** 1 Department of Ear, Nose, and Throat, Hopital du Valais, Sion, Switzerland; 2 Department of Otorhinolaryngology and Head and Neck Surgery, Centre Hospitalier Universitaire Vaudois (CHUV), Lausanne, Switzerland

**Keywords:** sinonasal inverted papilloma, Schneiderian papilloma, recurrence, endoscopic resection, Krouse classification

## Abstract

**Introduction** Sinonasal inverted papilloma (SNIP) is a rare benign epithelial tumor of the nasal cavity and paranasal sinuses that accounts for 0.4% and 4.7% respectively, of all tumors of this anatomical region.

**Objective** To analyze the outcomes after surgical resection of SNIP and identify the risk factors for recurrence in a Swiss tertiary center.

**Methods** We conducted a retrospective review of all cases of SNIP treated at the Lausanne university hospital between 2005 and 2018. All data available on the patients and tumors were collected for analysis. We studied the recurrence rate and looked for risk factors.

**Results** We included 57 patients with a mean age of 55.5 years. There were 46 primary cases (80.7%) and 11 recurrences (19.3%). Maxillary sinus was the most frequent location (33.3%). Approximately half of the patients (52.6%) presented with a T3 tumor according to the Krouse classification. The mean recurrence rate after surgery was of 17.5% and it was more frequent among the patients in the recurrence group (45.5%) than among the primary cases (10.9%), reaching statistical significance (odds ratio [OR] = 6.8; 95% confidence interval [95%CI]: 1.5–30.8; *p* = 0.0165). Most patients were treated endoscopically (94.7%). Frontal sinus location, higher Krouse stage, and combined approach seemed to increase the risk of recurrence, but without statistical significance.

**Conclusion** Difficult surgical access, as in the case of tumors located in the frontal sinus, higher stage of the disease, and previously operated cases carry the higher risk of incomplete resection and recurrence.

## Introduction

Sinonasal inverted papilloma (SNIP) is a rare benign epithelial tumor of the nasal cavity and paranasal sinuses that accounts for 0.4% and 4.7%, respectively, of all tumors of this anatomical region. 1 The reported incidence of SNIP worldwide ranges from 0.2 to 1.5/100 thousand patients per year. 2 It predominantly affects men, with a male-to-female ratio of 2-5:1. 1 The peak incidence appears to be in the fifth and sixth decades of life. 2

The precise etiology remains unclear and controversial. Some studies have suggested potential environmental risk factors 3 such as smoking, 4 and occupational exposure, 5 notably for patients who work in the construction, textile, printing, paper-making, and electronic industries. Some studies have shown molecular and cellular alterations which could be linked to the genesis of this tumor. Liu et al. 6 found an elevated expression of osteopontin and vascular endothelial growth factor (VEGF) in SNIP tissue compared with control tissue. Long-standing, chronic inflammation of the sinonasal mucosa is almost always associated with these tumors, as shown by Roh et al. 7 A viral origin is recognized and probably responsible for the 2% to 27% of cases which eventually degenerate into squamous cell carcinoma. 1 7 Even though human papillomavirus (HPV) is the recognized vector of these tumors, the 6/11 types known as non-carcinogenic are preponderant as compared with HPV (16/18), which probably explains the rather small percentage of cancerous degeneration. 8

In up to 23% of cases, SNIP remains asymptomatic. 9 Nasal obstruction, various degrees of hyposmia, and headaches are the most frequent symptoms. 9 On nasal endoscopy, it usually appears as a reddish-gray firm polyp with a characteristic strawberry aspect, often covered initially by a classic-looking grayish inflammatory polyposis. The ethmoid region, the lateral wall of the nasal fossa, and the maxillary sinus are described as the most frequent sites of the SNIP origin. 1 The Krouse classification is mostly used to describe the anatomical localization of SNIP based on radiological examination. 10 Though SNIP is benign in more than 90% of the cases, 2% to 23% of lesions may evolve into squamous cell carcinoma. 1 11 12 13

Surgery is required in cases of SNIP to enable complete resection of the tumor and its site of implantation to avoid recurrence, which is described in up to 25% of the patients. 11 Over the past three decades, the spectacular development of endoscopic surgical tools as well as of radiological diagnosis, which enable the precise location of the implantation of the SNIP, have significantly improved the management of these tumors, lowering the recurrence rate. 11 13 14

The risk factors for recurrence of SNIP described in previous studies 11 13 15 include surgical technique, 11 presence of HPV, 11 certain histological factors, 13 incomplete surgical resection, 15 revision surgery 15 and location of the tumor. 15

In the present study, we reviewed the experience of a swiss tertiary center, the Lausanne University Hospital, in the management of SNIP from 2005 to 2018. We have analyzed various patient or tumor factors and correlated them with treatment results, to identify potential risk factors for recurrence. Our data were then compared with the recent literature.

## Methods

After approval by the local ethics committee (Ethic Cantonal Commission, project-ID: CER-VD 2019–00591), we retrospectively reviewed the medical records of all patients treated surgically for SNIP from January 1st, 2005, to December 31st, 2018, in the Department of Otolaryngology and Head and Neck surgery at the Lausanne University Hospital, Switzerland. Age, gender, histopathology diagnosis, primary versus secondary case (recurrent case), radiological findings (computed tomography [CT], magnetic resonance imaging [MRI]), tumor extension and location, type of surgical approach (endoscopic, external or combined), use of image-guided navigation systems, complications, recurrence and duration of follow-up were collected.

Patients were included if they had been submitted to surgical treatment for a SNIP confirmed by histopathology and had undergone a minimum follow-up of 1 year. The exclusion criteria were insufficient follow-up (< 1 year), absence of research consent, or incomplete medical records.

The patients were separated into two groups: primary and secondary (recurrent) cases. The implantation of tumors and the involved sites were determined by reviewing the preoperative radiology and the surgical reports. The locations of the tumor were as follows: nasal cavity, maxillary sinus, ethmoid sinus, frontal sinus or sphenoid sinus. All patients included were retrospectively classified according to the Krouse staging system 10 by careful review of the preoperative CT and MRI scans. All surgeries were performed by experienced surgeons trained in endoscopic sinus surgery. The operative approach was chosen by the surgeon after clinical evaluation by nasal endoscopy and analysis of the radiological imaging. If the tumor extension and the site of the tumor implantation were adequate for an endoscopic approach, this surgery was preferred to any other approach. A combined approach was performed if necessary to achieve complete tumor resection with removal of the periosteum in the area of tumor implantation, according to Bugter et al. 16

The main outcomes were SNIP recurrence and time until recurrence, which were then compared with the surgical approaches, previous recurrences, tumor location and the Krouse staging. The results regarding the recurrence rate were expressed as mean ± standard deviation (SD) values, and the groups were compared with Mann-Whitney non-parametric tests using the GraphPad Prism 8 (Dotmatics, Boston, MA, United States). Values of *p* < 0.05 were considered statistically significant.

## Results

In total, 60 patients met the inclusion criteria; 3 were excluded due to missing information, and the remaining 57 patients were included in the present study. All were operated by experienced ear, nose, and throat (ENT) surgeons specialized in rhinology over a period of 13 years.

The patient demographics and clinical data are presented in Table 1 . There were 14 female and 43 male patients, with a mean age at diagnosis of 55.5 ± 16.26 (range: 15 to 91) years. The mean follow up was 65.6 ± 48.83 (range: 13 to 192) months. In total, 46 out of 57 patients (80.7%) were primary cases, and 11 (19.3%) were secondary (recurrent) cases.

**Table 1:** Patients demographics and clinical data ( *n* = 57)

Variable	n (%)
Gender	
- Male	43 (75.4)
- Female	14 (24.6)
Mean age in years (range)	55.5 (15–91)
Lesion side	
- Right	30 (52.6)
- Left	25 (43.9)
- Both	2 (3.5)
Primary case	46 (80.7)
Secondary case	11 (19.3)
Preoperative radiological imaging	
- CT scan	38 (66.7)
- CT scan + MRI	14 (24.6)
- No radiological imaging	5 (8.7)
Location	
- Nasal cavity	12 (21.1)
- Maxillary sinus	19 (33.3)
- Ethmoid sinus	17 (29.8)
- Sphenoid sinus	4 (7)
- Frontal sinus	5 (8.7)
Krouse stage	
- T1	12 (21.1)
- T2	13 (22.8)
- T3	30 (52.6)
- T4	2 (3.5)
Cancer	0
Surgical approach	
- Endoscopic	54 (94.7)
- Combined	3 (5.3)
- Bicoronal	1 (1.8)
- Caldwell-Luc	2 (3.5)
Surgical complications	2 (3.5)
- Cerebrospinal fluid leak	1 (1.8)
- Major hemorrhage	1 (1.8)
Perioperative neuronavigation	26 (45.6)
Mean follow-up in months (range)	65.6 (13–192)
Mean recurrence rate	10 (17.5)
Mean time until recurrence in months (range)	17.2 (3–48)
- Primary cases	4.8 (2–12)
- Secondary cases	23.8 (6–48)

**Abbreviations:** CT, computed tomography; MRI, magnetic resonance imaging. combined approach was only necessary in 5.3% (3/57) of patients (2 Caldwell-Luc and one bicoronal approach

Preoperative radiological imaging comprised CT scans alone in 38 cases (66.7%) and CT scan + MRI scan in 14 cases (24.6%). Overall, 5 patients (8.7%) did not have preoperative imaging, as they presented with a small SNIP well circumscribed in the nasal cavity.

The left- and right-side sinonasal cavities were almost equally involved (52.6 versus 43.9 respectively), and 2 patients (3.5%) presented bilateral involvement. The most frequent site of SNIP in the present series was the maxillary sinus, with 19/57 (33.3%) of the cases, followed by the ethmoid sinus (17/57; 29.8%), the nasal cavity (12/57; 21.1%), the frontal sinus (5/57; 8.7%), and the sphenoid sinus (4/57; 7%).

Each pre-operative CT scan and MRI scan available were reviewed independently by an experienced ENT surgeon to classify the extension of the tumor according to the Krouse staging system. In total, 12/57 cases (21.1%) were classified as stage T1, 13/57 (22.8%), as T2, 30/57 (52.6%), as T3, and 2/57 (2.3%), as T4. Patients were treated by endoscopic surgery alone in 94.7% of the cases (54/57). No patient was treated by external approach alone, and 5.3% (3/57) were treated by combined approach (endoscopic and external approaches). We performed 2 Caldwell-Luc procedures and 1 bicoronal approach.

An image-guided navigation system was used during surgery in 45.6% of the patients (26/57) whose tumor location was more anatomically complex. The recurrence rate was slightly higher among patients operated under navigation guidance (5/26; 19.23%) compared to the other group operated without this system (5/31; 16.13%).

The global recurrence rate was of 17.5% (10/57); among the primary cases, 10.9% (5/46) presented recurrence, and the rate was of 45.5% (5/11) in the group of patients operated for recurrent disease. This difference reached statistical significance (odds ratio [OR] = 6.8; 95% confidence interval [95%CI]: 1.5–30.8; *p* = 0.0165) ( Table 2 ). Patients operated endoscopically presented a lower recurrence rate (16.7%; 9/54) compared with the ones treated with a combined approach (33.3%; 1/3), but this difference was not significant ( *p* > 0.9). The Krouse staging did not predict the risk of recurrence in the present study ( Table 3 ).

**Table 2:** Recurrence rate according to the primary and secondary cases

	Recurrence rate: n(%)	*p* -value
Primary cases	5/46 (10.9)	0.0165
Secondary (recurrent) cases	5/11 (45.5)	

**Table 3:** Recurrence rate according to the Krouse stage

	Recurrence rate : n (%)	*p* -value
T1	1/12 (8.3)	Not significant
T2	2/13 (15.4)	Not significant
T3	6/30 (20)	Not significant
T4	1/2 (50)	Not significant

Regarding the different anatomical sites of implantation of the SNIP, none were significantly more at risk of recurrence; 60% (3/5) of the patients treated for a frontal sinus SNIP presented recurrence, as well as 17.7% (3/17) of those with the tumor in the ethmoid sinus, 15.8% (3/19) of those with a maxillary sinus SNIP, and 8.3% (1/12) of cases located in the nasal cavity. No recurrence was observed for tumors located in the sphenoid sinus. Even though it was the site of implantation with the highest rate of recurrence, the difference between the frontal sinus and the rest of the sinonasal locations was not significant ( *p* = 0.0525).

The mean time until recurrence was of 17.2 ± 51.49 (range 3 to 48) months. The mean time until recurrence among the primary cases was of 4.8 ± 10.82 (range: 2 to 12) months, and, among the secondary cases, it was of 23.8 ± 20.12 (range 6 to 48) months. The patients who presented recurrence (17.5%; 10/57) had a mean of 1.6 (SD value : 5.06) (range: 1 to 4) recurrences.

Severe complications were observed in two cases: one cerebrospinal fluid leak after resection of a SNIP located in the olfactory groove that required skull base plasty with fat, fascia lata, and mucosa, and one severe diffuse bleeding during the operation that required packing for a few days, concerning a patient treated with aspirin.

## Discussion

Multiple studies 11 13 15 17 have addressed the risk factors for recurrence of SNIP after surgery. Surgical technique, 11 the site of origin of the tumor (frontal), recurrent disease, 15 and Krouse stage 17 are often mentioned. Histopathological characteristics of the tumor, such as presence of HPV, 11 hyperkeratosis, elevated mitotic index, or severe epidermal hyperplasia have also been reported. 13

Peng et al. 11 described recurrence rates of 12.8%, 16.5%, and 12.6% for the endoscopic, external, and combined approaches respectively. These numbers are in line with those of a meta-analysis by Lisan et al., 17 which reported a rate of 13.8% for the endoscopic group and of 18.7% for the external approach group. In the present study, we found an overall recurrence rate of 17.5% (10/57), which is slightly higher than that of other reports, 11 15 probably due to a large proportion of recurrent cases.

Our recurrence rate for primary cases was of 10.9% (5/46), and of 45.5% (5/11) for secondary cases. This difference was statistically significant ( *p* = 0.0165). Previous studies have reported a higher recurrence rate after multiple surgeries. 18 Second recurrence after revision is frequent, with 50% of cases requiring a third operation. 16 These data were recently confirmed by a meta-analysis published in 2019. 11 In the present study, the second recurrence rate was of 40% (4/10), which is probably explained by the masking of the recurrence insertion point due to the distorted anatomy and the inflammation of the sinonasal mucosa due to previous surgery, as mentioned in the report by Bugter et al. 16

The initial tumor extension, as described in the Krouse stage, appears to play a role in recurrence rate, but the relationship is not always statistically significant. 12 19 We did not demonstrate an increased risk of recurrence for T3 Krouse stages compared with T2 (15.4%) or T1 (8.3%). This is in line with the study by Lisan et al., 19 who showed that a difference might be identified between advanced stage (T3-T4) and more localized disease (T1-T2) (OR: 1.51; 95%CI: 1.09–2.09) but no increased risk was found between Krouse stages T1 and T2 (OR: 1.14; 95%CI: 0.63–2.04) or between stages T3 and T4 (OR: 1.27; 95%CI: 0.72–2.26).

Both CT and MRI scans are considered standard in the preoperative work-up. In most cases, the combination of both enables the precise location of the SNIP implantation site. The combination of CT and MRI scans has shown a sensitivity of 94.6% and a specificity of 92.3% for the prediction of the SNIP implantation site, thus enabling optimal planning of the surgical procedure. 20 Only a limited number of patients (24.6%; 14/57), had been submitted to a CT scan and an MRI scan in the preoperative work-up in the present series; 38 patients had only undergone a preoperative CT scan and 5 patients presenting with nasal cavity lesions had not undergone imaging before surgery.

Due to the crucial importance of locating the implantation site of the SNIP, many studies have been progressively reporting new methods to improve both the preoperative location or perioperative identification of tumor margins. The use of optical coherence tomography and confocal laser scanning microscopy, as well as high-resolution microendoscopy using a specific contrast agent, proflavine, are being proposed for the differentiation of the SNIP from nasal polyps. 21 22 Li et al. 23 have also found that the Storz Professional Image Enhancement System (SPIES) is a rapid and non-invasive live-imaging technique that can detect the SNIP by examining the sinonasal mucosa and the submucosal and micro vasculature. These techniques have shown promising results, but their roles remain to be confirmed.

Neuronavigation was progressively introduced in our department, and it was used in 45.6% of the patients (26/57) who had either recurrent tumors or primary location in the frontal and the ethmoid sinuses. The use of navigation made almost no difference in the rate of recurrence, as 5/26 (19.2%) of the patients whose procedures involved navigation and 5/31 (16.1%) of those whose procedures did not involve navigation presented recurrence. As expected, reports in the literature encourage the use of navigation, as it seems to decrease overall recurrence. 20 Therefore, the use of navigation for most procedures became standard practice in our department in 2019. In most cases, preoperative MRI and CT scans enable the identification of the implantation site of the tumor, and they are used intraoperatively with neuronavigation.

One third (33.3%, 19/57) of our patients presented with a maxillary sinus implantation. For the few patients with a frontal presentation of the initial disease, the recurrence rate was high (60%; 3/5), but the numbers were too small to enable any statistically significant conclusions. But it is well established that this location is at higher risks of recurrence compared with all others. 12 13 17 19 24

As it appears to carry less morbidity, an endoscopic surgical excision of the SNIP is usually the preferred approach. It has been the case in our department for many years. 11 In our series, 94.7% (54/57) of the cases were treated by endoscopic surgery alone, and a combined approach was only necessary in 5.3% (3/57) of patients (2 Caldwell-Luc and one bicoronal approach). The two Caldwell-Luc procedures were performed because of recurrences on the lateral wall of the maxillary sinus, which was not endoscopically accessible. We probably could have avoided a Caldwell-Luc procedure by performing an endoscopic prelacrimal approach, but we did not perform this technique at that time. The bicoronal approach was performed due to a recurrence at the level of the supraorbital region and the lateral wall of the frontal sinus, which was not endoscopically accessible. None of the histopathology reports showed the presence of dysplasia or cancer in the resected tumors. Some hard-to-reach locations, such as the lateral wall of the frontal sinus or the anteroinferior wall of the maxillary sinus may require combined endoscopic and external approaches. 11 13

In the frontal sinus, a Draf III procedure usually enables the excision of the SNIP in most cases. 25 Depending on the anatomy of the supraorbital and/or frontomeatal recess, or in case of far lateral location of the tumor, a combined or external approach by frontal osteoplastic flap might be necessary. For the maxillary sinus, performing a modified endoscopic median maxillectomy or an endoscopic prelacrimal recess approach enables the resection of most tumors, regardless of their locations, without the need for a Caldwell-Luc approach. 11

As previously mentioned in the literature, 11 we noted a higher recurrence rate when a combined surgical excision was necessary (33.3%; 1/3) compared with the standard endoscopic approach (16.7%; 9/54). This difference was not statistically significant, and it is probably explained by differences in the extension and the location of the tumors in the two groups, justifying the choice for more extended surgery in one of them, thus increasing the relative risk of recurrence.

Most recurrences usually occur in the first 3 years following surgery. Bugter et al. 16 reported that 68% of the patients treated endoscopically presented recurrence in the first 24 months after treatment. Kim et al. 12 reported a mean recurrence time of 32.6 months. Long-term follow-up is generally recommended. 13 16 Our mean time until recurrence was of 17.2 ± 51.49 (range: 3 to 48) months. One patient presented a late recurrence 48 months after the first resection. The mean time until recurrence differed among the primary cases (4.8 ± 10.82 months; range: 2 to 12 months) and the secondary cases (23.8 ± 20.12; months; range 6 to 48 months). We found no rational explanation for this result, but it confirmed the need for a long-term follow-up.

Whereas many authors recommend endoscopic follow-up every three months for the first two years and then every six months for up to five years, it has been clearly established that MRI scans are only recommended for the investigations of proven recurrence or if endoscopic visualization of the sinus cavities is poor. 13 16 A CT scan can be performed if a surgical revision has to be planned.

The present study has certain limitations. First, it is a retrospective chart review, so the results could be biased due to the retrospective nature of the analysis. In addition, the present study included relatively few patients and the average follow-up was relatively short (< 5 years). Krouse staging was mostly completed using CT scans found in patient records by a single-blinded rhinologist. Only a minority of patients had an MRI scan (24.6%). This represents a significant bias to properly determine the Krouse stage. Actually, the CT scan does not always enable the accurate discrimination of inflammation and tumor invasion. Therefore, since 2019, we always plan preoperative MRI and CT scans in our department. They also enable a better surgery guidance for the resection of the tumor stalk, resulting in a reduction in the risk of recurrence. Surgery was performed by multiple surgeons with different levels of experience.

## Conclusion

Despite some limitations related to a retrospective chart review with inherent biases, the total SNIP recurrence rate was of 17.5% (10/57) in the present study.

Surgery for a recurrent tumor was the only factor which correlated with further recurrence. Frontal sinus implantation is a recognized difficult location to obtain perfect surgical clearance. This was confirmed by an increased risk of recurrence in the present study, without reaching statistical significance ( *p* = 0.0525). In total, 94.7% (54/57) of the patients were treated by endoscopic surgery, with only 5.3% (3/57) of the patients requiring a combined approach. With a recurrence diagnosed after 48 months in the present series, we confirm the need for a long-term follow-up for the patients treated for a SNIP, regardless of the initial location.

The development of more precise techniques to locate the SNIP insertion point and to guide complete excision regardless of the initial location is crucial. As most studies recently published have highlighted, the precision of the initial location of the SNIP, especially with a preoperative MRI scan, is important to improve the efficiency of the endoscopic treatment and decrease the recurrence rate.
